# Screening the anti infectivity potentials of native N- and C-lobes derived from the camel lactoferrin against hepatitis C virus

**DOI:** 10.1186/1472-6882-14-219

**Published:** 2014-07-03

**Authors:** Elrashdy M Redwan, Esmail M EL-Fakharany, Vladimir N Uversky, Mustafa H Linjawi

**Affiliations:** 1 Biological Science Department, Faculty of Science, King Abdulaziz University, P.O. Box 80203, Jeddah 21589, Kingdom of Saudi Arabia; 2 Therapeutic and Protective Proteins Laboratory, Protein Research Department, Genetic Engineering and Biotechnology Research Institute, City for Scientific Research and Technology Applications, New Borg EL-Arab, Alexandria 21394, Egypt; 3 Department of Molecular Medicine, USF Health Byrd Alzheimer’s Research Institute, Morsani College of Medicine, University of South Florida, Tampa, FL 33612, USA; 4 College of Applied Medical Sciences, King Abdulaziz University, P.O. Box 80203, Jeddah 21589, Kingdom of Saudi Arabia

**Keywords:** Camel lactoferrin, Proteolytic digestion, Purification, N- and C-lobes, Huh7.5 cells, Anti-HCV

## Abstract

**Background:**

Hepatitis C virus (HCV) infection represents a worldwide health threat that still needs efficient protective vaccine and/or effective drug. The traditional medicine, such as camel milk, is heavily used by the large sector of HCV patients to control the infection due to the high cost of the available standard therapy. Camel milk contains lactoferrin, which plays an important and multifunctional role in innate immunity and specific host defense against microbial infection. Continuing the analysis of the effectiveness of camel lactoferrin against HCV, the current study aimed to separate and purify the native N- and C-lobes from the proteolytically cleaved camel lactoferrin (cLF) and to compare their *in vitro* activities against the HCV infection in Huh7.5 cells in order to determine the most active domain.

**Methods:**

Lactoferrin and its digested N- and C-lobes were purified by Mono S 5/50 GL column and Superdex 200 5/150 column. The purified proteins were assessed through three venues: 1. To inhibit intracellular replication, HCV infected cells were treated with the proteins at different concentrations and time intervals; 2. The proteins were directly incubated with the viral particles (neutralization) and then such neutralized viruses were used to infect cells; 3. The cells were protected with proteins before exposure to the virus. The antiviral potentials of the cLf and its lobes were determined using three techniques: 1. RT-nested PCR, 2. Real-time PCR, and 3. Flow cytometry.

**Results:**

N- and C-lobes were purified in two consecutive steps; using Mono-S and Superdex 200 columns. The molecular mass of N- and C-lobes was about 40 kDa. cLF and its lobes could prevent HCV entry into Huh 7.5 cells with activity reached 100% through direct interaction with the virus. The inhibition of intracellular viral replication by N-lobe is 2-fold and 3-fold more effective than that of the cLF and C-lobe, respectively.

**Conclusion:**

Generated native N- and C-lobes from camel lactoferrin demonstrated a range of noticeably different potentials against HCV cellular infectivity. The anti-HCV activities were sorted as N-lobe > cLf > C-lobe.

## Background

The body fluids, organs and tissues of animals contain a number of natural antimicrobial agents that kill various microbes or inhibit their growth. One of these antimicrobial agents is lactoferrin (LF). LF is a multifunctional glycoprotein which found in milk and other secretions, and is known to play an active role in innate immunity. LF exerts its antimicrobial activity against various pathogens including fungi, bacteria, and viruses [1,2]. Lactoferrin is a member of the transferrin family and consists of two homologous lobes (N- and C-lobes). The two lobes are connected by a short “hinge” peptide and each lobe has one iron-binding site [3,4]. Each of the N- and C-lobes consists of about 345 amino acid residues and is made up of two domains N1, N2 and C1, C2, respectively. In addition to the iron binding ability, lactoferrin can bind to various kinds of cells [5], DNA [6], heparin and other glycosaminoglycans and lipopolysaccharides [7]. This ability to interact with different partners defines the multifunctional roles of LF which acts as an antibacterial [8] and antiviral agent [9], antioxidant, modulator of the immune and inflammatory responses [10,11], growth factor [12], and iron binding protein [11]. Camel LF consists of 689 amino acid residues and contains 17 disulfide bridges and four predicted glycosylation sites, one in the N-lobe and three in the C-lobe. The pattern of disulfide bonds in cLF is identical to those found in human and mare LFs, but the locations of predicted glycosylation sites are entirely different in cLF. There is a 70% identity between the whole sequence of cLF and that of other lactoferrins, but the first 50 residues of the N-terminus show an identity of less than 40%. Some residues, such as Pro418, Leu423, Lys433, Gln561, Gly629, Lys637, Arg652, and Pro592 related to movement of domains in the protein are different in cLF from those found in other LFs, indicating the possibility of specific structural differences [13].

LF exerts its activity towards both enveloped and naked viruses, and this activity was due to either inhibition of virus-host interaction, e.g., hepatitis B virus [14], herpes simplex virus (HSV) [15], and human cytomegalovirus [16], or resulted from the direct interaction between lactoferrin and the viral particle, e.g., hepatitis C virus (HCV) [17-21], human immunodeficiency virus (HIV) [22], and adenovirus [23]. HCV is an enveloped RNA virus belonging to the *Flaviviridae* family [24]. HCV infection is a major cause of chronic liver disease. In fact, more than 50% of individuals exposed to HCV develop a persistent infection associated with a chronic hepatitis, of which 7–16% will develop cirrhosis in the next 20 years following diagnosis [25]. HCV genotype 4 (HCV-4) is common in the Middle East and in Africa, where it is responsible for more than 80% of HCV infections. Although HCV-4 is the cause of approximately 20% of the 170 million cases of chronic hepatitis C in the world, it has not been the subject of comprehensive research [26]. In previous reports, we evaluated the anti-HCV potential of the full-length cLF and other camel milk proteins in hepatoma cell-lines [18,19,21]. Recent study was focused on the comparison of the anti-viral activities of recombinant versions of cLF (the full-length protein and its N-lobe) with the natural C-lobe, due to the fact that the recombinant C-lobe could not be properly expressed [20]. The goals of the current study were to enzymatically prepare, separate, and purify of the native N- and C-lobes from cLF, and then to screen the anti-infectivity potentials of these species in Huh 7.5 cells in comparison with that of full length cLF.

## Methods

### Lactoferrin purification

Camel milk was defatted and decaseinated as previously described by El-Fakharany *et al*., [19,27,28]. Skim milk was diluted with 50 mM Tris-HCl, pH 8.0 and sample containing 40 mg protein was applied to a cation-exchange Mono S-5/50 GL column (5 × 50 mm) pre-equilibrated with 50 mM Tris HCl, pH 8.0. Then, the column was washed with the same equilibration buffer to remove impurities. The elution was carried out with 50 mM Tris HCl, pH 8.0 and NaCl gradient from 0.0 to 1.0 M at flow rate of 1.0 ml/min and the fraction size of 1.0 ml/fraction. The fractions were screened by SDS-PAGE and their cLF content were assayed by ELISA using monoclonal antibodies against hLF, then pooled and concentrated, then a sample containing 0.4 mg protein was applied to a size-exclusion Superdex 200-5/150 column (5 × 150 mm) pre-equilibrated with 50 mM Tris HCl, pH 8.0. Elution of cLF was carried out with the same equilibration buffer at a flow rate of 0.3 ml/min and the fraction size of 0.5 ml/fraction.

### Proteolytic hydrolysis of camel lactoferrin

Iron-saturated lactoferrin was prepared by the procedure of Mazurier and Spik [29] and Khan *et al*. [13]. Then, the solution of iron-saturated protein was passed through a Sephadex G-25 column (PD-10 Desalting Columns) to remove the excess of ferric chloride. For hydrolysis of lactoferrin, the following enzymes were used: proteinase K (20 unit/mg), trypsin from bovine pancreas (14923 units/mg), pepsin from bovine pancreas (700 units/g), α-chymotrypsin from bovine pancreas (59.3 unit/mg) and papain (140 unit/mg). The enzymes were added to iron saturated cLF solution at a ratio 1:50. The hydrolysis reaction was performed at 37°C for 0.5 and 1 h, then the hydrolyzed mixtures were kept at -20°C.

### Purification of N-lobe and C-lobe

The N- and C-lobes were purified after hydrolysis of cLF using proteinase k at a ratio 1:50 and incubated at 37°C for 1 h. The N- and C-lobes were separated by loading the digested cLF to a cation-exchange MonoS 5/50 GL column (5 × 50 mm) and eluted with 50 mM Tris HCl, pH 8.0 using a NaCl gradient from 0.0 to 1.0 M at flow rate of 0.7 ml/min. The isolated peaks were further purified on a gel filtration column of Superdex 200-5/150 in 50 mM Tris-HCl (pH 8.0). Identification of the purified N and C-lobes lactoferrin were confirmed by N-terminal amino acid sequencing.

### Infected serum samples

For all infection experiments, we utilized PCR-HCV positive serum samples of genotype 4a from Egyptian patients from our out-clinics (after approval by the ethical committee of the Genetic Engineering and Biotechnology Research Institute) as described previously. The peripheral blood leukocytes from whole blood were obtained from health volunteers after introduce our ideas and experiments for them. A clear consent was obtained from each patient or volunteer (we will be using his or her blood samples in our *in vitro* experiments and we will publish the data without disclosing his/her name).

### Cell culture, media and endotoxin determination

Huh7.5 derived cells permissive for the HCV entry were kindly donated by Prof. Charles Rice at the Rockefeller University (New York, NY 10065–7919, USA). Cell line preservation, culture media, and protocols for running the cultured cells were used as previously published [30-32]. The endotoxin content was checked to avoid its pyrogenic effects on the cell-culture system [33]. All purified proteins used were free of endotoxin (data not shown). Lactoferrin and its N- and C-lobes concentration were estimated with two methods [34,35].

### Cytotoxicity assay of cLF, N-, and C-lobes

Before treatment with cLF, N- or C-lobe, Huh 7.5 cells were incubated at 37°C for 2 days in a 96-well plate. The medium was refreshed with new supplemented medium containing 0.5 or 1.0 mg/ml of protein and cells were incubated for 4 days at 37°C and 5% CO_2_. Twenty μl of MTT solution (5 mg of MTT per 1 ml PBS) were added to each well and incubated at 37°C for 3-5 hours to allow the MTT to metabolize. Formazan crystals were dissolved by DMSO and quantified by measuring the absorbance of the solution at 595 nm. The relative cell viability (%) was calculated by (A) test/ (B) control × 100% [36-39].

### Anti-HCV activity of cLF, N-, and C-lobes

To test the anti-HCV potential of cLF, N-, and C-lobes, we examined the protein - virus interactions with either the cells (protection activity) or viral particles (neutralization activity) or in infected cells (treatment). First, to examine protection activity, Huh 7.5 (1.0 × 10^5^) cells were plated in 24-well plate and incubated for 24 h. The purified cLF, N-, or C-lobe were added to Huh 7.5 cells at concentration of 1.0 mg/ml, and then incubated for 60 min at 37°C. After removing unbound proteins by washing three times with 1 ml of PBS, 1 ml of medium containing 50 μl HCV-infected serum (5 × 10^6^ copies/ml, HCV genotype 4) [40,41] was added, and the cells were incubated at 37°C for 90 min. The cells were washed three times with PBS and cultured for 7 days at 37°C and 5% CO_2_. Second, to examine neutralization activity, 50 μl of HCV-infected serum were pre-incubated with the purified proteins in 1 ml media at 4°C for 60 min. Subsequently, the mixtures were added to the Huh 7.5 (1.0 × 10^5^) cells cultured in the 24-well plates and incubated at 37°C for 90 min. Cells were then washed three times with PBS and further incubated for 7 days at 37°C and 5% CO_2_[21,30]. Positive and negative control cultures were included. The cells were washed three times to eliminate debris and dead cells by using RPMI 1640 supplemented media, and then prepared for RT-PCR [19-21,32].

### Evaluation of the ability of cLF, N-, and C-lobe to treat the HCV infected Huh 7.5 cells

Huh 7.5 (1.0 × 105) cells were plated in a 24-well plate and cultured for 24 h at 37°C and 5% CO_2_, then infected with 50 μl of HCV-infected serum in 1 ml RPMI 1640 media. After incubation for 2 days, free HCV was removed by washing cells three times with culture media, and then the purified cLF, N-, or C-lobe proteins were added at concentrations of 0.25, 0.5, 0.75, 1.0 and 1.25 mg/ml and the cells were incubated for 4 days at 37°C and 5% CO_2_. After washing the cells three times from debris and dead cells by PBS, viral components were traced by RT-PCR [19-21,30].

### RT nested PCR

Samples of RNA from Huh 7.5 cells were prepared and RT nested PCR, both were performed according to El-Fakharany *et al*. [19-21,30,39]. Synthesis of the cDNA and first PCR reaction were carried out in a single-step PCR reaction using Ready-To-Go RT-PCR beads (Amersham Pharmacia Biotech) in two stages as described below. First stage was conducted at 42°C for 45 min followed by 98°C for 10 min using 1CH forward primer and P2 reverse primer. Second stage protocol was as follows: 1 min at 94°C, 1 min at 55°C, and 1 min at 72°C for 35 cycles. The second round PCR (nested PCR) was performed using reverse primer D2 and forward primer F2 and the protocol was similar to the first round PCR reaction [19,20,30]. To control false detection of negative-strand HCV RNA and to known variations in PCR efficiency, specific control assays and rigorous standardization of the reaction were employed. A Rulc plasmid was included as internal control during the amplification process. The final amplified DNA samples were electrophoresed through 3% agarose gel and ethidium bromide was used to visualized 174 bp for HCV and 374 bp of Rluc.

### Real time PCR

Briefly, HCV RNA was extracted from Huh 7.5 cells by INSTANT Virus RNA Kit according to the manufacturer’s instructions. Amplification of HCV RNA in samples and standards was measured by RoboGene HCV RNA Quantification Kit using the Rotor-Gene real time PCR machine and the report was generated by Rotor-Gene Q Series Software 1.7 (Build 94) Copyright© 2008 Corbett Life Science, a QIAGEN. The relative activity (%) was calculated as [(A) count of positive control – (B) count of tested protein]/(A) count of positive control × 100% [39].

### Intracellular tracing of HCV

Flow cytometric analysis was used to elucidate the antiviral activity of the cLF and its lobes against HCV. The cells were washed three times from debris and dead cells using RPMI 1640 media or 1.0× PBS. Intracellular labeling was performed by indirect immunofluorescence. Cells were centrifuged and cell pellets were washed twice with 1.0 × PBS containing 1% normal goat serum, cells were incubated with 4% paraformaldehyde for 10 min and 0.1% Triton X-100 in Tris HCl buffer pH 7.4 for 6 min. After washing three times with 1.0 × PBS, cells were incubated with monoclonal antibody (P26664, clone C7-50, Pierce-Thermoscientific) against HCV core (1:1,000) at room temperature for 1 h [42]. The cells were stained with fluorescein-conjugated goat anti-mouse (KPL, USA) incubated at 4°C for 30 min. After being washed, cells were suspended in 2 ml PBS and analyzed by Flow cytometry [18,30].

### Statistical analysis

Data were analyzed by Student’s t-test. All experiments were repeated three times and each value represents the average of three determinations. Data are shown as means ± SEM (the standard error of the mean) of three independent experiments. A *P*-value of ≤0.05 was considered statistically significant.

## Results

### Camel lactoferrin purification

**Figure 1:**
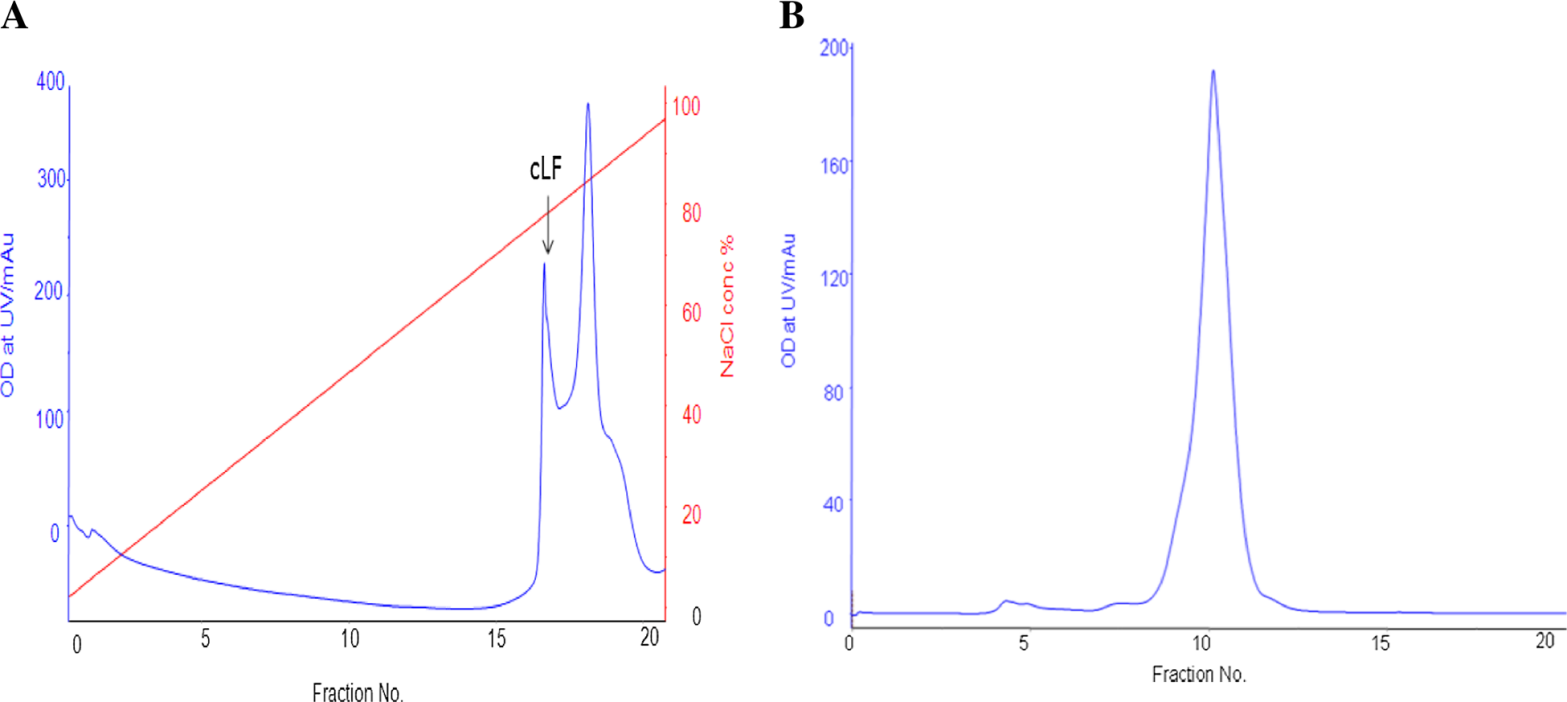
**Camel lactoferrin purification by Mono S-5/50 GL.** A typical elution profile of camel milk lactoferrin on a Mono S-5/50 GL column **(A)** pre-equilibrated with 50 mM Tris-HCl buffer, pH 8.0 at flow rate1.0 ml/min and fraction size of 1.0 ml/fraction and the fractions containing cLF were pooled, concentrated and then loaded on a Superdex 200-5/150 column **(B)**.

**Figure 2:**
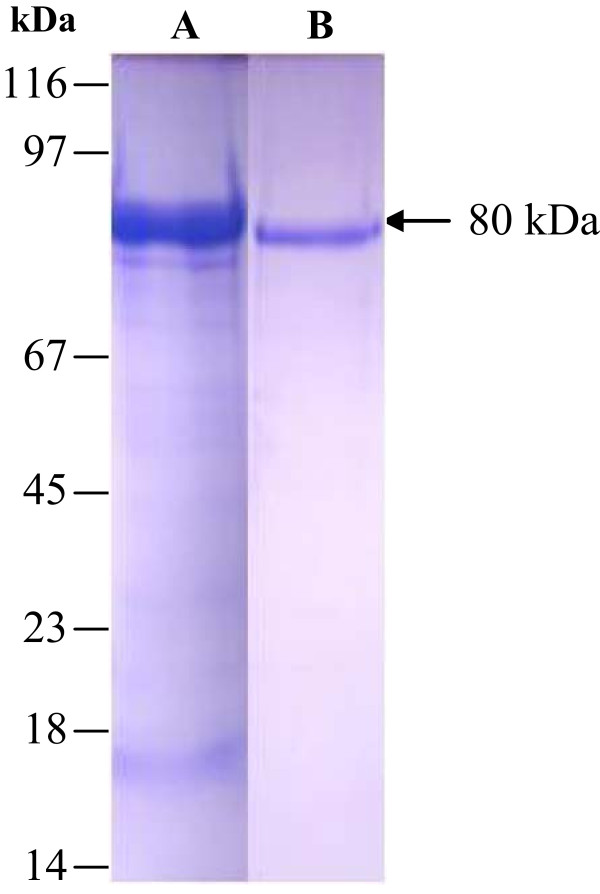
**SDS-PAGE of lactoferrin profile.** 12% SDS-PAGE of camel lactoferrin during purification on Mono S-5/50 GL column **(A)** and Superdex 200-5/150 column **(B)**.

Camel lactoferrin was purified from skimmed milk by chromatography on a cation-exchange MonoS 5/50 GL column and eluted by 0.0-1.0 M NaCl gradient. The peak containing cLF (Figure 1A) was analyzed by SDS-PAGE and then titrated by ELISA (data not shown). The concentrated fractions were applied into a size-exclusion Superdex 200 5/150 column (Figure 1B). Single discrete band has been visualized on 12% SDS polyacrylamide gel of the protein and estimated to be 80 KDa as shown in Figure 2.

### Hydrolysis of camel lactoferrin

**Figure 3:**
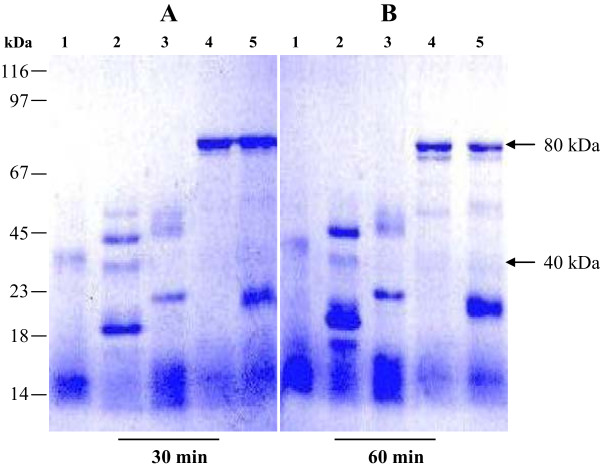
**Non-reducing PAGE of mild hydrolysis lactoferrin.** SDS-PAGE of camel lactoferrin under non-reducing condition after mild hydrolysis for 0.5 h **(A)** and 1.0 h **(B)**: lactoferrin hydrolyzed by proteinase K (lane 1), lactoferrin hydrolyzed by trypsin (lane 2), lactoferrin hydrolyzed by pepsin (lane 3), lactoferrin hydrolyzed by chymotrypsin (lane 4) and lactoferrin hydrolyzed by papain (lane 5).

The results obtained indicated that the proteinase K hydrolyzes lactoferrin giving a clear band at 40 kDa synchronized with a band corresponding to a substantial quantity of low molecular mass peptides (<14 · 4 kDa) after 0.5 or 1 h from incubation of enzyme and substrate as shown in Figure 3. However, trypsin activity produced four major fragments of approximately 50, 45, 35 and 23 kDa, together with small molecular mass peptides after 0.5 h of incubation of the enzyme and lactoferrin. Three major fragments of approximately 45, 35 and 23 kDa, in addition to small molecular mass peptides after 1.0 h of incubation (Figure 3) have been produced by trypsin digestion. Pepsin-driven proteolysis produced three major fragments of approximately 49, 46 and 30 kDa besides the small molecular mass peptides after 0.5 and 1.0 h of incubation with lactoferrin (Figure 3). The above three enzymes have the ability to complete hydrolysis of camel lactoferrin after 0.5 and 1.0 h from the starting reaction at the 1:50 ratio. On the other hand, chymotrypsin and papain enzymes failed to hydrolyze camel lactoferrin completely after 0.5 h or 1.0 h of incubation with lactoferrin at the 1:50 ratio (Figure 3).

### Separation and purification of N- and C-lobes

**Figure 4:**
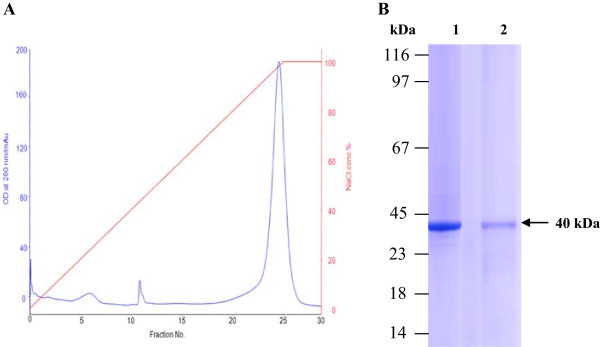
**Lactoferrin N-lobe purification profile. (A)** A typical elution profile of N-lobe lactoferrin on a Mono S 5/50 GL column pre-equilibrated with 50 mM Tris-HCl buffer, pH 8.0 at flow rate 0.7 ml/min and fraction size of 1.0 ml/fraction. **(B)** 12% SDS-PAGE of the N and C lobes of camel lactoferrin during purification on a Sephadex G-50 column. Lane 1, N lobe, lane 2, C lobe.

N- and C-lobes of cLF were purified from cLf proteinase K digestion reaction using a cation-exchange Mono S 5/50 GL column. The results showed that the native N-lobe was eluted at 0.9 M NaCl while the C-lobe did not bind to resin (Figure 4A). The fractions containing N- and C-lobes were further purified on a size-exclusion Superdex 200 column. The purity of the two lobes was analyzed by 12% SDS-PAGE where both species corresponded to a molecular mass of ~40 kDa (Figure 4B). Protein identities of the purified N- and C-lobe were confirmed by the N-terminal sequencing, with N-terminal peptide sequences of Ala20, Ser21, Lys22, Lys23, Ser24, Val25, Arg26, Trp27 and Leu340, Arg341, Arg342, Ala343, Glu344, Val345, Val346, Trp347, respectively.

### Cytotoxicity effect of cLF, N-, and C-lobes

Cell proliferation was assayed by MTT method using Huh 7.5 cell-line to exclude any possibility that the elimination of the viral particles was caused by reducing the viability of the cells. The results revealed that both 0.5 and 1.0 mg/ml of N-lobe failed to exhibit any significant effect on the Huh7.5 cells viability after 7 days of incubation. However, both cLf and C-lobe induced a reduction in Huh 7.5 cell-viability after 7 days of incubation at concentrations of 0.5 and 1.0 mg/ml (Table 1).

**Table 1:** Cell viability of Huh 7.5 cells by MTT method

**Concentration**	**Protein**			
	**Control**	**Lactoferrin**	**N-lobe**	**C-lobe**
0.5 mg/ml	100 ± 0.0477	92 ± 0.0312	97. ± 0.0424	91 ± 0.0374
1.0 mg/ml	100 ± 0.0248	87 ± 0.0441	96 ± 0.0441	88 ± 0.0278

The values are expressed as percentage of control cell viability.

### Anti-HCV activity of the cLF, N-lobe and C-lobe

**Figure 5:**
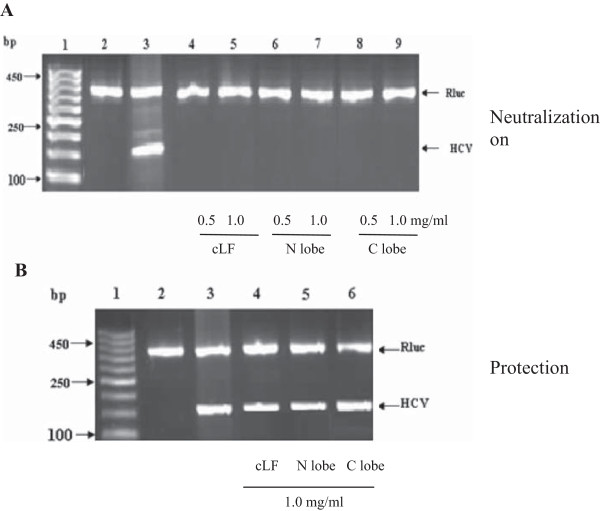
**Activity of cLF, N-lobe and C-lobe against HCV entry into Huh 7.5 cells by. (A)** HCV particles were pre-incubated with purified proteins prior to infection (neutralization). **(B)** Huh 7.5 cells were treated with purified proteins prior to infection with HCV (protection). Lane 1, DNA ladder; lane 2, negative control; lane 3, positive control. Lane 4-9, Proteins at different concentrations, as indicated under the gel graph. The upper arrow pointed the Rulc which served as internal control, while the lower arrow pointed the positive amplified 174 bp of HCV.

Our results indicated that camel lactoferrin, and its N- and C-lobes are able to completely inhibit the HCV entry into the Huh 7.5 cells. Two sets of (1.0 × 10^5^) Huh 7.5 cells were cultured, in duplicate; one of the cultures was inoculated with HCV infected sera pretreated with cLF, N- or C-lobe (0.5 or 1.0 mg/ml). The inoculated cells were cultured for seven days. At different proteins concentration used, a band of 174 bp was not amplified (Figure 5A), which indicate that there is no detectable HCV molecules. The RT-nested PCR was used to amplify the 174 bp of 5’ end of HCV noncoding sequence in comparison to the positive and negative control. The other cultures were treated with cLF, N- or C-lobe (1.0 mg/ml), for 60 min, and then infected with HCV for 90 min. The inoculated cells were cultured for seven days. All proteins at all concentration used failed to protect the cells from the HCV entry (Figure 5B).

### Evaluation of cLF, N- and C-lobes ability to treat the HCV-infected cells

**Figure 6:**
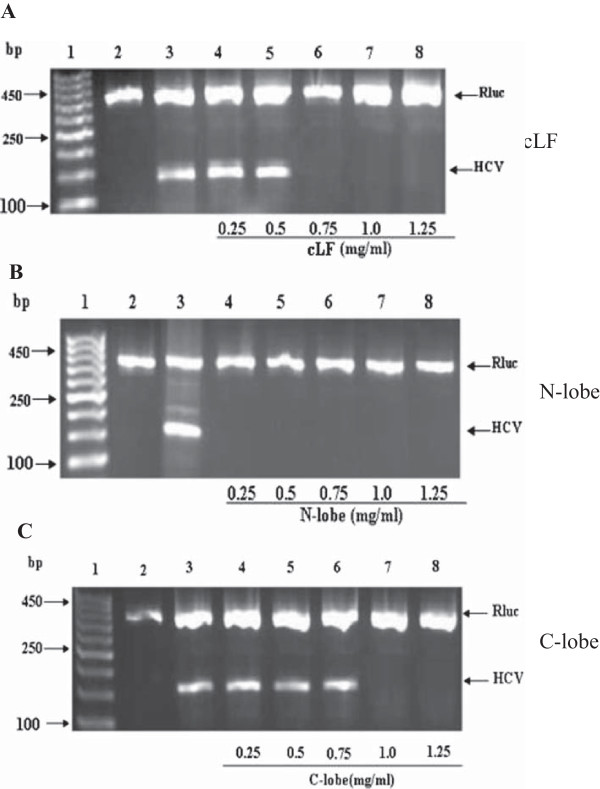
**Intracellular inhibition of HCV replication in infected Huh 7.5 cells by cLF (A), N-lobe (B) and C-lobe (C).** HCV infected Huh 7.5 cells were treated with cLF, N-lobe or C-lobe for 96 h. RT nested PCR was performed to amplify viral RNA segments. Lane 1, DNA ladder; lane 2, negative control; lane 3, positive control; lane 4-8, proteins at different concentrations, as indicated under the gel graph. The upper arrow pointed the Rulc which served as internal control, while the lower arrow pointed the positive amplified 1740 bp of HCV.

Camel lactoferrin, and its N- and C-lobes at concentrations of 0.25, 0.5, 0.75, 1.0 and 1.25 mg/ml were investigated for their *in vitro* ability to inhibit the viral replication inside the infected Huh7.5 cells (treatment). Inhibition of viral replication was detected by amplification of viral RNA segments using the RT-PCR technique. Camel lactoferrin could inhibit the intracellular HCV replication at concentrations starting from 0.75, 1.0 and 1.25 mg/ml after 4 days (Figure 6A). However, N-lobe could inhibit the HCV replication at all concentrations used (0.25-1.25 mg/ml) after 4 days (Figure 6B). Whereas the C-lobe has revealed its inhibition potentials on the HCV replication only at concentrations of 1.0 and 1.25 mg/ml after 4 days of incubation (Figure 6C). This may indicated a marked superiority of N-lobe over the full-length cLf and C-lobe in treatment of infected cells, with N-lobe being three- and four-fold active than the full-length cLf and C-lobe, respectively.

### Real time PCR evaluation of the antiviral activity of the full-length cLF and its N- and C-lobes against HCV

Based on the HCV copy number calculations, we concluded that the full-length cLF and its N- and C-lobes at concentrations of 0.5, 1.0 mg/ml are able to completely inhibit the HCV entry into Huh 7.5 cells with the relative activity of 100%. On the other hand, cLf, N-lobe, and C-lobe could protect the Huh7.5 cells against HCV entry with the relative activity of 69.39%, 76%, and 29%, respectively (Table 2). The activity of cLF against intracellular HCV replication was steadily increased with increase in concentration from 0.25 mg/ml to 0.75 mg/ml, approaching about 51% to 95% after the first treatment, whereas the activity became 100% (at 1.0 and 1.25 mg/ml) after the first dose and (at 0.75 mg/ml) after the second. The second dose of cLf could reduce the intracellular HCV load from 6.290.580 to 0.0 IU/ml at concentrations of 0.25 and 0.5 mg/ml, respectively (Table 3). The N-lobe of cLf could inhibit viral replication from the first dose at 0.25 mg/ml showing the relative activity of 99%, but it reaches a 100% relative activity at 0.5 to 1.25 mg/ml without any need for second treatment (Table 3). The activity of the native C-lobe against intracellular HCV replication is increased from 24% at 0.25 mg/ml to 100% at 1.25 mg/ml after the first treatment, while its activity became 37% at a 0.25 mg/ml, 77% at 0.5 mg/ml and reached 100% at 0.75 and 1.0 mg/ml only after the second treatment (Table 3).

**Table 2:** **Detection of HCV RNA in infected Huh 7.5 cells**^**a**^

**Protein**	**Type of experiment**	**Protein conc. (mg/ml)**	**Calc. conc. (IU/ml)**	**Relative activity (%)**
Control	Control	Positive	339500	0.0 ± 0.01
	Control	Negative	0.0	100 ± 0.0
Lactoferrin	Neutralization	0.5	2380	99.29 ± 0.027
		1.0	0.0	100 ± 0.0
N-lobe	Neutralization	0.5	0.0	100 ± 0.0
		1.0	0.0	100 ± 0.0
C-lobe	Neutralization	0.5	3430	98.98 ± 0.011
		1.0	620	99.81 ± 0.025
Lactoferrin	Protection	1.0	103910	69.39 ± 0.032
N-lobe	Protection	1.0	80790	76.20 ± 0.029
C-lobe	Protection	1.0	240700	29.10 ± 0.015

^a^Real time PCR data for camel lactoferrin, N-lobe and C- lobe activity against HCV. Huh 7.5 cells served as negative control, and infected Huh 7.5 cells with HCV served as positive control.

**Table 3:** **Detection of HCV RNA in infected then treated Huh 7.5 cells**^**a**^

**Protein**	**Protein conc. (mg/ml)**	**Calc. conc. (IU/ml)**	**Relative activity (%)**
Control	Positive	53910	0.0 ± 0.01
	Negative	0.0	100 ± 0.0
Lactoferrin (first dose)	0.25	26110	51.56 ± 0.038
	0.5	12430	76.94 ± 0.02
	0.75	2210	95.59 ± 0.045
	1.0	0.0	100 ± 0.0
	1.25	0.0	100 ± 0.0
N-lobe	0.25	520	99.03 ± 0.023
	0.5	0.0	100 ± 0.0
	0.75	0.0	100 ± 0.0
	1.0	0.0	100 ± 0.0
	1.25	0.0	100 ± 0.0
C-lobe (first dose)	0.25	40790	24.33 ± 0.032
	0.5	24070	55.35 ± 0.046
	0.75	13320	75.29 ± 0.033
	1.0	1030	98.08 ± 0.041
	1.25	0.0	100 ± 0.0
Lactoferrin (second dose)	0.25	6290	88.33 ± 0.012
	0.5	580	98.92 ± 0.03
	0.75	0.0	100 ± 0.0
C-lobe (second dose)	0.25	33950	37.02 ± 0.026
	0.5	12370	77.05 ± 0.011
	0.75	0.0	100 ± 0.0
	1.0	0.0	100 ± 0.0

^a^Real time PCR data for in vitro cLF, C- and N-lobe activity-treatment against HCV-infected Huh 7.5 cells. Huh 7.5 cells served as negative control, and infected Huh 7.5 cells with HCV served as positive control.

### Intracellular tracing of HCV

**Figure 7:**
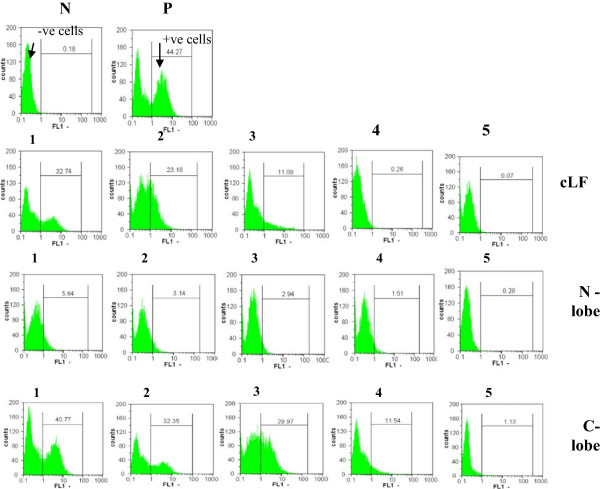
**Results of the flow cytometric analysis of intracellularly labelled HCV-infected Huh 7.5 cells.** HepG2 cells served as negative control (N) and Huh 7.5 cells infected with HCV served as positive control (P). Cells were stained intracellularly with anti-rabbit antibody conjugated with FITC. Effect of cLF, N lobe and C lobe at concentrations of 0.25, 0.5, 0.75, 1.0 and 1.25 mg/ml (1, 2, 3, 4 and 5, respectively) on the HCV infected Huh 7.5 cells.

The flow cytometric analysis results have been confirmative for the RT-nested-PCR and real time PCR results. Figure 7 indicates that the high fluorescence signal scan profiles could be detected in case of cLF at 0.25 and 0.5 mg/ml and C-lobe at 0.25, 0.5 and 0.75 mg/ml. Whereas the N-lobe at all concentrations used could not detect any fluorescence signal in flow cytometry scan profile. However, the low fluorescence signal in flow cytometry scan profiles could be detected with C-lobe at concentration of only 1.0 mg/ml (Figure 7).

## Discussion

Lactoferrin shown to have strong inhibitory activities against pathogens, including fungi, bacteria and viruses. In a recent study, LFs isolated from different species (sheep, goat, camel, alpaca, elephant, human, and bovine) were effective in inhibiting *E. coli* 0157:H7 infection and cLF was found to be the most active among other lactoferrins [43]. The antiviral activities of cLF span a broad spectrum of viruses, such as cytomegalovirus, polyomavirus, HSV, HIV, HBV, HCV, simian rotaviruses, and adenovirus [44]. HCV is a major cause of parenterally transmitted non-A, non-B hepatitis [45], and infection with HCV is one of the leading causes of chronic liver disease worldwide [46]. The infection with this virus is still without efficient therapeutic or preventive agents [42,47]. The prevalence of HCV infection has increased during the recent years, with an incidence of 3% in the world's population, and in some countries like Egypt, the incidence is up to 15% [48] and reach up to 20% in some Delta governorates [49]. However, there have been relatively few studies that dealt with the patients infected with HCV genotype 4, and combination therapy trials (interferon and ribavirin) for these patients have not demonstrated promising efficacy [49].

The current study attempted to separate and purify N- and C-lobes from the proteolytically digested camel lactoferrin and to evaluate potential inhibitory effects of these proteins on HCV entry into the Huh7.5 cells. Huh7.5 are normal HCV-susceptible cells [50,51]. Separation of the N-lobe from the C-lobe is a useful approach to study the cLF peptide map and the potential activity of each lobe alone against different pathogens allowing determination of which lobe has more distinguished anti-HCV activity. In addition, the molecular weight reduction to 40 kDa (or less) may minimize the possible antigenecity. Our results revealed that camel lactoferrin could be purified in two steps, and its molecular mass was estimated to be 80 KDa, using SDS-PAGE. This result is consistent with that of Kappeler *et al*., [52] and is slightly higher than that of Elagamy *et al*. [53], who estimated that molecular mass of camel milk lactoferrin is 79.5 kDa. These variations in the molecular mass may reflect the milk source-dependent differences in the glycosylation patterns of the lactoferrins used in different studies.

The N- and C-lobes were prepared by enzymatic hydrolysis of iron-saturated cLF. Different protein fragmentation profiles were generated by different enzymes (trypsin, pepsin, chymotrypsin, papain and proteinase K). The best profile was produced by the proteinase K at ratio 1:50 after 60 min, where digested lactoferrin mostly produced polypeptides of 40 kDa and to less extend some low molecular mass peptides of <14 · 4 kDa. The digested cLF was fractionated using a cation-exchange Mono S column. Both lobes were further purified by gel-filtration Superdex 200 column and identified as the C- and N-lobes based on their N-terminal sequences as previously reported [21]. The molecular weight of produced lobes were estimated to be 40 kDa, which was congruent with those of Khan *et al*. [13] who used proteinase K to digest cLF and isolated N- and C-lobes with estimated molecular weight of 40 kDa, and Sharma *et al*. [54] who obtained N- and C-lobes from buffalo LF. Both studies used the manual ion exchange and gel filtration chromatography. Over nearly three decades of work on generation of the N- and C- lobes which included both the proteolysis as well as cloning efforts, the proteolysis of lactoferrin with proteinase K continues to be the most effective and productive way of production of pure N- and C-lobes which are not only used as antimicrobials but also were utilized in structural studies leading to the crystallization and structure determination of the C-lobe [55].

The cytotoxicity of cLF and its N- and C-lobes was tested by the cell MTT proliferation assay. The highest cytotoxic effect was established for C-lobe (91%, 88% at 0.5 and 1.0 mg/ml, respectively) followed by intact cLf (92%, 87% at 0.5 and 1.0 mg/ml, respectively), whereas N-lobe possessed the lowest cytotoxic effects.

Although the C-lobe is expected to be structurally similar to the N-lobe based on the amino acid similarity of 35% [56], their antiviral potencies are quite different as our results have demonstrated. The structure of the cLF C-lobe has not been determined as of yet [56]. In addition, cloning the LF in different expression systems would produce protein with glycosylation profile different from that of the native LF, which may be reflected a different mode of action.

Native cLF or its N- and C-lobes are able to neutralize the HCV particles with relative efficiency of about 100%, suggesting that both the N-lobe and the C-lobe possess functional domains sufficient for the recognition of the E1 or E2 proteins in the HCV envelope or blocking the virus receptors on the cell surface. *In vitro*, the N-lobe was more potent than cLF and C-lobe in treatment of the HCV-infected cells. Here, it was able to inhibit the HCV replication inside infected Huh7.5 cells with the relative activity around 100% at concentrations from 0.25 to 1.25 mg/ml after 4 days. This inhibitory activity of cLF seems to be in agreement with the previous study, which used cLF to inhibit HCV (genotype 4) entry into human PBMCs [18]. The potency of native N-lobe does not significantly different from recombinant N-lobe [20], which may indicate the lack of influence of glycosylation profiles variation on the antiviral activity of these species. Redwan and Tabll [18] study confirmed the HCV entry to the host cells by RT-nested-PCR and by the indirect intracellular immunostaining of HCV E1 by flow cytometry, whereas in current study we used RT-nested PCR, real time PCR, and flow cytometric analysis as the HCV infectivity assays. HCV real-time RT-PCR has a great potential for a wide application in both basic and clinical studies. This technique, with its high sensitivity, increased specificity, broad range of detection capability, simplicity, and high reproducibility is particularly useful for screening large numbers of specimens and measuring viral loads to monitor the disease progression and effect of anti-HCV therapy [57]. Also, flow cytometry is an advantageous analytical technique in comparison with other methods due to its ability to i) measure the fluorescence intensity per cell, ii) count the infected cells directly, iii) examine several replicates simultaneously, and iv) elevate the accuracy by statistical analysis [18,30,39]. Therefore, combination of these techniques provides a very reliable approach to show that so the full-length lactoferrin and its lobes possess a differential potential against intracellular HCV replication.

Beleid *et al*., [58] had evaluated five synthetic peptides derived from the C-lobe of human lactoferrin (residues 600-632) with the length ranging from 17 to 33 residues against the HCV cellular entry. These peptides have variable α-helical content. Two out five peptides were able to specifically bind to the viral E2 and prevent the HCV from the cell entry. Sorting these peptides based on their helicity and binding activity, the authors concluded that the binding affinity increases with increase in the helical content. For example, peptide-5 (helicity 71%) was 10-folds better E2 binder than peptide-3 with 38% helicity. However, the authors did not compare their designed peptides with the native lactoferrin and/or its entire C-lobe. Same peptide (600-632) of the C-lobe (C-s3-33) derived from human lactoferrin in three forms (C-s3-33), (C-s3-33)_2_, and (C-s3-33)_3_ have been studied in details on PH5CH8 cell line [59]. It was found that although the tandem repeats of (C-s3-33)_3_ enhanced the ant-HCV activity compared with the monomeric C-s3-33 form, but the antiviral activity of the three forms was still several fold weaker than that of the original human lactoferrin [59]. Besides, the aforementioned lactoferrin peptides, many other peptides have been isolated from lactoferrin and characterized against several pathogens. Three of these peptides (LF1-11, lactoferrampin, and lactoferricin) were shown to possess the most antimicrobials activity and therefore were studied in much detail. These peptides were mostly derived from the N-lobe of lactoferrin and possess some intrinsic structural characteristics, such as hydrophobicity, cationicity, helical propensity, and high *pI* values (>9), which are important determinants of their antimicrobial potency [60]. According to the Expasy protein calculator, the N-lobe, C-lobe and intact camel lactoferrin have *pI* values of 9.15, 7.48, and 8.63, respectively. This, perhaps, explains why the N-lobe of camel lactoferrin was shown to have a superior potency over the intact lactoferrin and its C-lobe in the three experimental models analyzed in the current study.

Generally, the lactoferrin is considered as a rich source for cationic and amphipathic peptides, which may use against wide range microbes [56]. These peptides are derived from the lactoferrin polypeptide chain and are releases upon the proteolysis of the lactoferrin with different enzymes, which can be developed into clinically useful lead molecules for antimicrobial therapeutics [56,61]. Simulation of stomach enzymatic condition effects on camel lactoferrin using Expasy enzymatic cutter gave 616 hits, of which 8 peptides (≥10 residues) and 11 peptides (7-9 amino acids) have the *pI* values ranging from 5.99 to 10.06. This finding may agree with the results of the current study and previous reports [62], which demonstrated that taking bovine lactoferrin orally reduce the verimia in HCV patients. These finding also agree with our recent results (manuscript in preparation) showing that when camel milk was consumed in specific regime for 4-5 months by chronic HCV patients, the viral load was noticeably decreased whereas the patients’ performance was significantly improved.

Since the blood circulating lactoferrin is cleared by the liver [63], lactoferrin interacts with hepatocytes, which bind large amount of Lf with high affinity (*K*_d_ approx.20-100 nM). T-lymphocytes and monocytes bind and internalize bound Lf much less efficient in comparison with the hepatocytes, which can take up ~5000 Lf molecules per cell, per second [64] through the clatherin-mediated endocytosis [65]. Previous reports found that the tryptically generated C-lobe of lactoferrin was bound and internalized by hepatocytes in a manner similar to that of the native lactoferrin [64]. Although the tryptic N-lobe fragments were able to bind to the hepatocytes with higher affinity in a Ca^2+^-dependent manner, they were poorly internalized by hepatocytes as compared to the internalization of the C-lobe and the native lactoferrin [64]. This means that to see the effects on the HCV particles, one should use more N-lobe than C-lobe and the native lactoferrin. On the other hand, this capability to bind and internalize the lactoferrin and its lobes differently may explain our results on the different intracellular anti-HCV potency of these three proteins.

**Figure 8:**
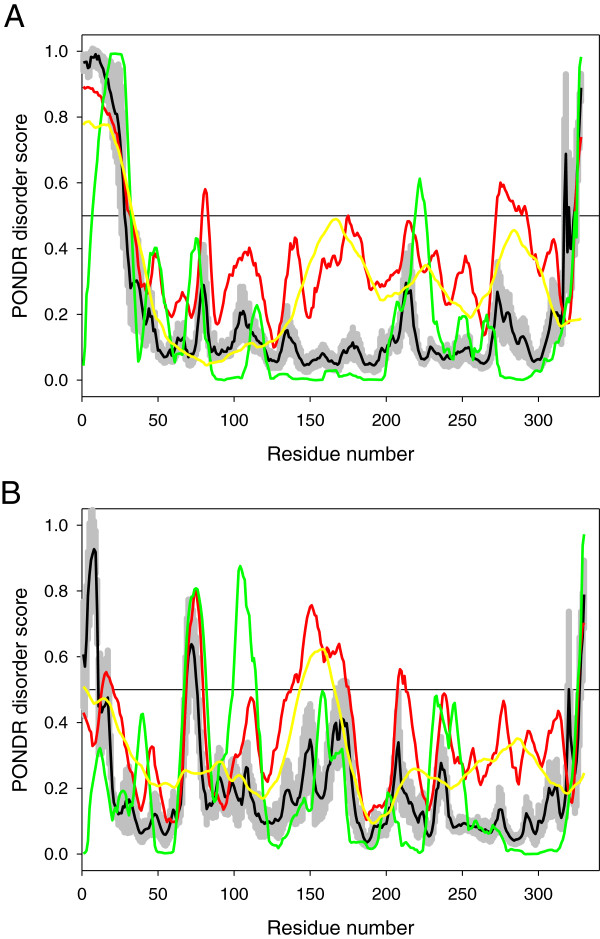
**Intrinsic disorder evaluation of N-lobe, C-lobe of camel lactoferrin.** Evaluation of intrinsic disorder propensities of the N-lobe (**A**, residues 25-352) and C-lobe (**B**, residues 364-693) of camel lactoferrin (UniProt ID: Q9TUM0). The per-residue intrinsic disorder propensity of these lobes was evaluated using four members of the PONDR family, PONDR® VLXT (green lines), PONDR® VSL2 (red lines), PONDR® VL3 (yellow lines), PONDR-FIT (black lines); sections with scores higher than 0.5 correspond to disordered regions. Light gray shadow around the PONDR-FIT lines corresponds to the standard errors of disorder prediction by PONDR-FIT.

It also very likely that this difference in the biological activities of cLf and its lobes can be determined by the divergence in some of their structure-related characteristics. This hypothesis is supported by Figure 8, which represents the result of disorder propensity evaluation in the N- and C-lobs of camel Lf by a set of disorder predictors of the PONDR family. PONDR® VLXT is sensitive to local sequence peculiarities, and therefore is useful for predicting short disordered regions that become structured when they interact with other proteins [66]. PONDR® VL3 is better for proteins that are experimentally known to be 100% disordered and therefore is a useful tool for finding long disordered regions [67], whereas PONDR® VSL2 is statistically better for proteins containing both structured and disordered regions [68]. Finally, PONDR-FIT is a metapredictors which is statistically not different from VL3 for fully disordered and fully structured proteins, and is slightly better than VSL2 when both structure and disorder are present [69]. Figure 8 clearly shows that the disorder propensities of the N- and C-lobe are quite different, with the N-lobe being expected to be noticeably less disordered and/or flexible than the C-lobe. It is likely that this difference in the disorder propensity is related to the functional differences reported for these two lobes. Particularly, more rigid structure of the N-lobe combined with its higher net charge (+11 versus +1 in C-lobe) might represent a more suitable platform for interaction with binding partners involved in the inhibition of the HCV infection. In fact, intrinsically disordered regions and regions with increased flexibility were shown to be common in viral proteins [70-73], where they play a number of important roles in virus infectivity [73-77], virus evolution [78], as well as in various functions of viral proteins, including their interactions with the host cell proteins [73,76,77,79], and in the antiviral defense of the host organism [80].

Our results demonstrated that there are at least three potential venues for the cLf and its lobes to serve as antiviral agents. Although the full-length cLF and its lobes possess different efficiencies, they all can protect the Huh7.5 against HCV infection, neutralize the intracellular HCV replication, and treat the infected Huh7.5 cells. HCV entry to the host cell is a complex, multistage process requiring action of four receptors, fusion, and endocytosis. HCV fusion depends on the E1 and E2 proteins, viral dose, and occurs within a specific pH range. Is the lactoferrin and/or its lobes iron sequestration or release have a role in the pH changes within the microenvironment around the cells during HCV cellular entry? It is still unclear how the lactoferrin and/or its lobes act against viral particles. Several mechanisms of action have been suggested for the LFs antiviral activities. Two of these mechanisms are 1) direct interaction between the viral molecules and LF, and 2) LF interaction with the cellular glycosaminoglycans (more specifically, the heparin sulfate, HS) of the viral receptors. The most widely accepted hypothesis is the LF-viral receptor pathway [81]. The binding of LF and HS prevents the first contact between HCV and the host cell and therefore prevent the infection.

The ubiquitous heparan sulfate proteoglycans (HSPGs), which are widely distributed on mammalian cells, have been identified as potential targets for a number of viruses [82-85], such as herpes virus [86], hepatitis C virus [87], dengue virus [88], human immunodeficiency virus type 1 [55], foot and mouth disease virus [55], human papillomavirus [89], and hepatitis B virus [90]. It has been shown that HSPGs acts as primary binding sites, promoting viral docking and facilitating subsequent interaction of viruses with the specific cellular receptors [91]. Importantly, lactoferrin can interact with the host cells before they are infected with hepatitis B virus (HBV), and such interaction of LF with the cell surface proteins can block the viral adhesion to the target cells [14]. Bovine lactoferrin (bLF) inhibits the viral entry into hepatocytes and lymphocytes via neutralizing the virion and blocking the invasion of the cell, but showed no antiviral activity after HCV internalization into the cells [92]. However, N-lobe of camel lactoferrin mostly exerts its action on HCV through the intracellular pathway, therefore supporting the hypothesis that antiviral activity of LF depend on the virus type, protein structure, and bioassay system used [92,93]. In line with this view, camel lactoferrin and its C-lobe showed weaker potency than N-lobe in the case of the HCV infection, whereas the inhibition of influenza virus heagglutination and cell infection of all major influenza virus subtypes were entirely attributed to the C-lobe of bovine lactoferrin only [94]. Finally, third way of the antiviral action of LF is the virocidal venue, which has been recently proposed based on the protein cationicity and α-helical structure of lactoferrins [58,65,95,96].

## Conclusion

Our results demonstrated that the camel native lactoferrin and its N- and C-lobes have divergent inhibitory action against *in vitro* HCV (genotype 4a) entry into the Huh7.5 cells. This inhibition was not restricted only to the neutralization of the viral molecules, but the activity was extend to the suppression of the intracellular viral replication. Native cLF its N- and C-lobes also possess some protective effects on the cells themselves. These remarkable anti-HCV activities of camel Lf and its lobes require additional work to understand how they work during their extra- and intracellular antiviral action. Additional work is also needed to find safe ways of N-lobe delivery *in vivo*. It is also clear that the action of the lactoferrion and its lobes on the HCV cannot be completely understood without looking deeply into their structures.

## Competing interest

Authors declare that they have no competing interest.

## Authors’ contributions

EME, conduct tissue culture, viral screening research, made protein purifications and all immunoassays; EME and EMR wrote the draft of manuscript, tabulated the data, and collected some references; MHL contributed with new reagents/analytical tools, help in manuscript revision, data organization, data analysis, EMR, design research, manuscript finalizing in its final form. EMR and VNU constructed the figures, revised the draft, and put together the final version. All the authors read and approved the final manuscript. This work is dedicated to the spirit of Dr. Nezar A Redwan.

## Pre-publication history

The pre-publication history for this paper can be accessed here:

http://www.biomedcentral.com/1472-6882/14/219/prepub
